# When remediating one artifact results in another: control, confounders, and correction

**DOI:** 10.1007/s40656-023-00606-2

**Published:** 2024-01-11

**Authors:** David Colaço

**Affiliations:** Munich Center for Mathematical Philosophy, LMU Munich, Munich, Germany

**Keywords:** Experiment, Artifact, Experimental control, Confounder

## Abstract

Scientists aim to remediate artifacts in their experimental datasets. However, the remediation of one artifact can result in another. Why might this happen, and what does this consequence tell us about how we should account for artifacts and their control? In this paper, I explore a case in functional neuroimaging where remediation appears to have caused this problem. I argue that remediation amounts to a change to an experimental arrangement. These changes need not be surgical, and the arrangement need not satisfy the criterion of causal modularity. Thus, remediation can affect more than just the factor responsible for the artifact. However, if researchers can determine the consequences of their remediation, they can make adjustments that control for the present artifact as well as for previously controlled ones. Current philosophical accounts of artifacts and the factors responsible for them cannot adequately address this issue, as they do not account for what is needed for artifact remediation (and specifically correction). I support my argument by paralleling it with ongoing concerns regarding the transparency of complex computational systems, as near future remediation across the experimental life sciences will likely make greater use of AI tools to correct for artifacts.

## Introduction

Scientists aim to remediate artifacts in their experimental datasets. These artifacts result from factors in an experimental arrangement that are distinct from the target factor or variable. While philosophers of science have taken interest in artifacts and the factors responsible for them (Colaço, 2018; Schickore, 2019; Craver & Dan-Cohen, 2021; Feest, 2022), their remediation has not been addressed. However, if an artifact is not remediated, it undercuts the evidential use of data collected in this experiment.

In this paper, I introduce an account of artifacts and their control by exploring a problem that can arise: the remediation of one artifact can result in another artifact, where the data would not have the second had researchers not attempted to remediate the first. I explore a case in which the remediation of an artifact caused this problem: head motion in fMRI research. Studies show that the motion of subjects’ heads causes “spurious but systematic correlation structures” in fMRI datasets (Power et al., 2012). However, remediation of head motion resulted in fMRI datasets with novel respiratory artifacts (Fair et al., 2020). Respiration is a well-known factor in fMRI studies (Friston et al., 1996; Zaitsev et al., 2015), but the remediation of head motion resulted in respiration confounding researchers’ studies. Why did this happen, and what does it tell us about artifacts and their control?

The answer to this question, I will argue, is that remediation is an intervention that changes an experimental arrangement, but one should not presuppose that it surgically affects only the intended factor in the arrangement, nor should one presuppose that the arrangement itself satisfies the criterion of causal modularity (Woodward, 2005, p. 329).^1^ If the intervention is not surgical or the arrangement is not modular, the consequence of this intervention will not be a change to only the intended factor(s). Rather, other factors, even known factors, might now result in an artifact. Determining the consequences of a remediation method is best achieved via determining how this remediation works. This entails that remediation is not solely tied to factors responsible for an artifact. Potentially, it can affect any other factor in the arrangement, including previously applied remediation methods. The position that I defend comes with the implications that currently characterized experimental probes into factors responsible for artifacts (Schickore, 2019) need not be sufficient, while meeting specified challenges in designing experiments without the presence of these factors (Feest, 2022) need not be necessary.

I support my answer by showing that a certain subset of remediation methods, those that are automated and involve the use of algorithms, might not be structurally transparent (Creel, 2020), capturing that researchers are unable to understand how they bring about a result when they are incorporated into an experimental arrangement. Beyond supporting my position, this insight also provides us a look forward to a future of remediation in the experimental life sciences. Given that the advance of AI tools will likely result in an increase of the use of these tools for remediation, I reflect on how these tools should be trained and interpreted, giving us the best chance of avoiding future cases like the one described in this paper.

In Sect. 2, I introduce artifacts, the factors responsible for them, and three methods for remediating them. In Sect. 3, I detail my case study of head motion artifacts in fMRI research. I discuss how the correction of head motion artifacts led to novel respiration artifacts. In Sect. 4, I answer why the remediation of one artifact might result in another, drawing on surgical remediation methods and the causal modularity of the arrangement. Further, I defend the need for probes that support causal reasoning. In Sect. 5, I strengthen my position by exploring the potential future of artifact remediation and the issues it is liable to face if my recommendations are not taken into consideration.

## Artifacts and their remediation

An artifact is a stable aspect of data resulting from one or more factors in an experimental arrangement that are distinct from the target factor or variable. Colloquially, artifacts result from “unwanted” contributions to data. My account pulls ideas from Craver and Dan-Cohen’s account of artifacts (or artefacts),^2^ where an artifact is “a systematic deviation in the value of D [data] produced by causes A of D other than T [the target of the study] that break or weaken the indicative correlation of D with T” (2021, p. 21). It also pulls ideas from Feest’s account, where “artifacts are the conclusions of unsound inferences, when researchers mistakenly think that they have reliable data” (2022, p. 13).

### What artifacts are

First, artifacts are in data. Thinking about artifacts in this way is unlike thinking of them as human creations (Baker, 2004), but it matches how scientists often use this term.^3^ To avoid confusion, this paper addresses what might be called data or experimental artifacts, rather than something like Aristotelian artifacts.

Second, artifacts are stable. They are not random error that can be eliminated through repeated sampling. Rather, they are systematic error, or the regular, predictable discrepancies between an observed value and the expected value for a given target (Woodward, 1989, p. 460). Systematic error, sometimes called bias (Kahneman et al., 2021), persists even if researchers resample data via their experimental arrangement. The fact that artifacts are stable is why researchers must control for them via remediation or other alterations to the arrangement rather than washing them out with more data from the same arrangement.

Third, what researchers aim to investigate is not what is responsible for the artifact. Rather, a separate factor or set of factors, included in the experimental arrangement but otherwise orthogonal to the target of the study, is responsible (Colaço, 2018). This highlights that artifacts are interest-relative, consistent with Craver and Dan-Cohen’s discussion of a “deviation” stemming from causal factors aside from the target factor or variable.

Fourth, the factors that are responsible for artifacts are present in an experimental arrangement. I define ‘experimental arrangement’ as the set of all factors that influence the data outcome of a given experiment. As I use the term in this paper, the arrangement includes *both* data production and data processing. Artifacts can result from any factor or set of factors (aside from the target) in the arrangement. For instance, artifacts can result from data production, such as metal moving near MRI instrumentation, or data processing, such as data realignment.

My discussion of factors responsible for artifacts relates my analysis to Schickore’s discussion of *confounders* in her framework addressing experimental control in the life sciences. Schickore characterizes confounders as factors that “muddle (disrupt, distort) the relation between independent and dependent variables in an experimental situation,” (2019, pp. 211–212). A confounder in this sense is the kind of factor that can be responsible for an artifact. Schickore introduces the idea of a “confounder repertoire,” which “encompasses the kinds of factors that are likely to interfere with an actual experimental inquiry” and tabulates the kinds of factors that might result in artifacts in a study (2019, p. 212). Though Schickore does not explicitly address artifacts, Feest’s account links the two, where artifacts are data and confounders are “unwanted causal factors in the experiment, i.e., by variables that might distort the processes required in order for the experimental data to serve as evidence for a specific claim” (2022, p. 13). This relation contrasts with Craver and Dan-Cohen’s account, where artifacts are a subclass of confounds (2021, p. 21). For this paper, I talk of data artifacts, and I reserve the term ‘confounders’ for factors in the arrangement. This is unlike Craver and Dan-Cohen’s account. Terminology aside, what is important is that we distinguish aspects of data (what I call artifacts) from factors of an experimental arrangement (what I call confounders).

With artifacts characterized, the issue they present becomes salient. Researchers collect data that serve as evidence for their targets, which requires a reliable connection between the two (Woodward, 2000; Feest, 2022). While random error, the sum of nonrecurring factors in an experimental arrangement, is unavoidable, it does not threaten this connection given sufficient sampling. The factors responsible for artifacts, by contrast, do threaten this connection, and the identification of an artifact reveals this threat. A confounder responsible for an artifact is akin to an undercutting defeater (Pollock, 1987): its identification is a reason for denying that researchers would not have acquired the dataset they acquired unless they had detected their target.^4^ This supports Schickore’s characterization of confounders as muddling relations between variables but also the idea that “the presence of confounders does not necessarily lead to erroneous results; confounders may confuse, and no result can be obtained” (2019, p. 212). If the dataset contains an artifact, researchers might think that their target is responsible for their data and make unwarranted inferences (Feest, 2022).

### The control and remediation of artifacts

Philosophical accounts address the need to “control” for artifacts. In her analysis of the logic of experimental inference, Feest states the challenges that control should meet, such as “ensuring that the experimental manipulation does indeed causally affect (only) the variable of interest,” that “the experimental measurement does indeed (only) measure the variable presumed to be impacted by the variable,” and “that there are no additional causal factors distorting the experimental effect” (2022, p. 9). While these challenges are, Feest admits, difficult to meet in practice, researchers should aim to meet them on her account. Meeting this set of challenges is a “necessary condition” for concluding that “a particular subject matter is supported by experimental data” (Feest, 2022, p. 13). Likewise, in her analysis of experimental control, Schickore gives us a picture of the first step of controlling for confounders. This is achieved, on this account, through experimental controls that serve as probes. These “control-experiments” involve making a context-dependent identification that there is a confounder present in the experimental arrangement (Schickore, 2019, p. 211). Her account includes diagnostic probes, where “the task is to figure out where to look for confounders,” as well as determinative probes, where “the task is to determine what confounders are indeed present in a given experimental situation” (Schickore, 2019, p. 213). The results of these probes contribute to the confounder repertoire.

I specify three methods by which scientists can remediate an artifact once it has been identified. The first and most intuitive method is to *prevent* an artifact: eliminate factors responsible for an artifact from the experimental arrangement. If a factor is absent from the experimental arrangement, it cannot result in an artifact. For instance, if one wishes to remove head motion artifacts from fMRI data, one can restrain the subject’s head (Zaitsev, 2015, p. 894). If there is no head motion, there will be no data resulting from head motion.

The second method is to *reduce* an artifact: limit the detection or measure of a factor responsible for an artifact, thus limiting its influence on the data collected in the arrangement. For example, head motion artifact reduction can be achieved by shorter fMRI scan times, limiting the time in which the subject’s head motion can be detected by the scanner (Zaitsev, 2015, p. 894). Alternatively, researchers can time their recordings to miss a factor in the arrangement, such as the subject’s heartbeat (Zaitsev, 2015, p. 896). Shielding also reduces an artifact, as it makes the detection instrumentation less sensitive to factors in the arrangement that would otherwise affect the dataset.

The third method is to *correct* for an artifact. Following estimation of the artifact, its presence in data is compensated via changes in data processing. This method corresponds to what researchers have in mind when they “remove” an artifact from their dataset (e.g., Power et al., 2012, p. 2147), which reflects the idea that correction is a means of making a dataset look the way it would have looked had there been no artifact. Given that correction does not change data production, this method can be used long after production is complete, allowing for retroactive remediation of artifacts.

Though outwardly varied, these methods share something in common. They all involve changing data production or processing in an experimental arrangement, thereby changing its data outcome. We can therefore think of them as interventions on factors in the arrangement: prevention and reduction on factors of data production, and correction on data processing. In fact, one could “control” artifacts by these remediation methods, by running multiple, distinct studies (Culp, 1994), or simply by developing a new experiment. In all cases, the shared element is that controlling for artifacts requires differences in the factors of the arrangement. This fact hints at the answer to why remediating one artifact can result in another. Remediation is an intervention that changes the arrangement. However, the consequence of this intervention need not be a change to only the intended factor(s), either due to the nature of the intervention or to the arrangement upon whose factors are intervened.

While this paper addresses remediation generally, I focus on correction for three reasons. First, researchers might opt for correction when it is more ethical, practical, or convenient than prevention or reduction. One way in which it can be more practical or convenient is that, when using correction, researchers need not meet or even attempt to meet the challenges stated by Feest: when correction is successful, researchers need not ensure or even aim to ensure that their manipulation only affects, or their measure only measures, the relevant variable. Second, the fact that a factor is still a part of the arrangement entails that it still can affect the dataset when correction methods are used. Third, correction can be achieved automatically rather than manually. The rapid advance in the power of AI technologies lends support to the idea that correction will become ever more common in the future of the experimental life sciences.

## Artifacts in rs-fcMRI research

If researchers use resting-state functional connectivity magnetic resonance imaging (rs-fcMRI) to study a subject’s brain, it can be a problem if this subject moves their head during their scan. This is because head motion can result in artifacts in fMRI data.^5^ How do researchers attempt to control for these head motion artifacts? I discuss how these artifacts problematize rs-fcMRI findings, and how their correction led to a distinct artifact in rs-fcMRI data.

Before discussing the case, let me review a few fundamentals of functional neuroimaging. fMRI measures blood oxygen level dependent (BOLD) signals. BOLD signals are measurements of the magnetic difference between oxygen-rich and oxygen-depleted blood cells, which indicate that oxygen is consumed by neural tissue shortly after its activation. Thus, fMRI indirectly measures brain activity. rs-fcMRI has additional properties. First, the subjects are not performing a task. Rather, they are at wakeful rest: researchers ostensibly measure the subject’s spontaneous brain activity. Second, researchers correlate the activity of areas across the brain, as these correlations suggest functional networks. For these reasons, resting-state functional connectivity scans take longer than task-dependent scans.

### Head motion

rs-fcMRI initially showed promise in studying functional connectivity networks. Based on studies using this technique, researchers reported that there is a developmental maturation pattern in children: they exhibit strong short-range and weak long-range connections in their brain that weaken and strengthen, respectively, over the time of development (Fair et al., 2008). In addition, researchers reported that individuals with neurodevelopmental disorders exhibit a greater number of strong short-range connections and weak long-range connections when compared to neurotypical individuals of the same age (Cherkassky et al., 2006). These findings suggest that development and disorders correlate with the functional connectivity of one’s brain.

These promising findings were questioned following the determination of head motion artifacts. Van Dijk and colleagues report that “comparisons between groups of individuals with subtly different levels of head motion yielded difference maps that could be mistaken for neuronal effects in other contexts,” and “these effects are important to consider when interpreting variation between groups and across individuals” (2012, p. 431).^6^ This report suggests that head motion is a confounder when measuring functional connectivity: head motion results in artifacts that are like the results predicted by researchers’ connectivity hypotheses (Van Dijk et al., 2012, p. 431).^7^ Likewise, Power and colleagues report that “many long-distance correlations are decreased by subject motion, whereas many short-distance correlations are increased” (2012, p. 2142). Head motion thus correlates with the short- and long-range connections that appear in rs-fcMRI data independently of brain development or neurodevelopmental disorders.

Following determination of this artifact, this promising research was undermined. Upon attempts to remediate this artifact, researchers found that the “maturation pattern usually disappears when head motion is taken into account” (Hughes, 2012, p. 3). These reports led one researcher, Steve Peterson, to colorfully remark that “it really, really, really sucks. My favorite result of the last five years is an artifact” (reported in Hughes, 2012, p. 3). Research on neurodevelopmental disorders did not fare any better. While these artifacts do not prove that all functional connectivity research on brain development or disorders is faulty, Damien Fair notes that these artifacts are “going to require folks to reanalyze their data, controlling for these new ways of examining motion” (reported in Hughes, 2012, p. 3).

Compounding researchers’ frustrations is that they were aware of head motion, and it was in the confounder repertoire (Friston et al., 1996). Researchers thought that they had corrected for head motion artifacts with realignment. Realignment adjusts the images taken of the brain so that the scan is in the same place and position despite head motion. Researchers match scanned images to a reference image, which involves moving and rotating the scanned images. Following this match, the differences between the scanned and reference images are quantified. As Power and colleagues put it, “since subjects move during scans, it is standard practice to estimate the position of the head in space at each volume of the data and to realign all volumes using rigid body transforms” (2012, p. 2142).

While existing realignment methods once seemed adequate, they are ineffective in these cases: “these changes in rs-fcMRI” are not “adequately countered by some common functional connectivity processing steps” (Power et al., 2012, p. 2142). This ineffectiveness stems from the relations amongst the correction method, MRI, and head motion itself. Realignment affects the relative place and position of brain images, but it “does not correct intensity changes resulting from disruption of the physical principles underlying MRI” (Power et al., 2012, p. 2142). The magnetic gradients that rs-fcMRI depends on are disrupted by head movement, which realignment does not correct. To put the point somewhat simplistically, it is not just the motion of the head in space that is the problem; it is also magnetic properties of the stuff in the head that is moving. Even in tandem with regressions performed on motion estimates, artifacts persisted in rs-fcMRI data. Head motion’s disrupting influence on magnetic gradients, paired with the fact that head motion includes everything from an acute jerk to a chronic shake, make remediating these artifacts challenging.

Because their existing methods were inadequate, researchers developed new methods for correcting head motion artifacts in rs-fcMRI datasets. Power and colleagues introduced a method they call “scrubbing,” where “two indices of data quality that can be used to flag frames of suspect quality, creating temporal masks of the data” are “combined in various ways to produce a final temporal mask, which specifies frames to ignore when performing calculations upon the data” (Power et al., 2012, p. 2146). This “final temporal mask,” or templated cutoff for data quality, is based on indices that relate to alignment and image intensity. If a frame of neuroimaging data exceeds the cutoffs specified by either index reflected in the temporal mask, this frame is removed from the dataset, hence why it is called “scrubbing.”

Scrubbing incorporates earlier realignment and regression methods. It also involves cutoffs for exceeding indices, automatically excising suspect frames of data from the dataset. These facts convey that scrubbing is more complex than earlier correction strategies, and they exemplify the changing standards for correcting head motion artifacts. Many of the correction methods introduced after the determination of head motion are automatic, using algorithms that have greater complexity than anything involved in realignment. For instance, methods like “denoising” (Xu et al., 2014) and “smoothing” (Scheinost et al., 2014) automatically correct head motion artifacts in datasets, with mixed to positive outcomes (Goto et al. 2013).

### Respiration

Novel correction methods seemed to make progress on removing head motion artifacts from rs-fcMRI datasets. However, their use created a new problem. As Fair and colleagues put it, “respirations contaminate movement estimates in functional MRI and… respiration generates apparent head motion not associated with functional MRI quality reductions” (2020, p. 1). A subject’s respiration is a separate factor in the experimental arrangement. Further, researchers could control for respiration before the advancement of new head motion correction methods (Murphy et al., 2013), allowing researchers to keep respiration from resulting in an artifact. Nonetheless, respiration is a confounder in the arrangement when any of these new head motion remediation methods are included.

Like head motion, respiration is multifaceted, involving body motion and the flow of gases. Breathing rates, stability, and intensity vary amongst individuals but also vary depending on the state of the individual, such as when they are nervous. Scrubbing, denoising, and smoothing methods were unable to include the effect of respiration but also remove the effect of head motion on this dataset. And, because subjects breathe more than they move their heads, these correction methods greatly changed the data, making these datasets much less useful.

Recognizing this problem, Power and colleagues began to collect more data on respiration during fMRI scans by having subjects wear abdomen belts that measure respiration variation, force, and breath cycle (2020). They then compared this respiration data to fMRI data in attempts to measure how various factors of respiration correlate with the rs-fcMRI dataset. This strategy was in the effort of developing an algorithm that can be used to correct for respiration. Their findings suggest that respiration can be modeled in datasets when deploying this algorithm, but certain so-called “deep breaths” are often missed by this algorithm (Power et al., 2020). Together, these findings raise concerns about how researchers might address respiration in rs-fcMRI data, further compounding worries about the use of these methods for correcting head motion.

## Why remediating one artifact can result in another

The story of neuroimagers’ attempts to correct for head motion and later respiration captures the tribulations researchers face when they attempt to remediate an artifact. In this case, both artifacts resulted from factors in the experimental arrangement. Head motion undercuts the reliability of rs-fcMRI datasets for addressing questions about connectivity differences between children and adults as well as between neurotypical and patients with neurodevelopmental disorders. With head motion, which is “subtly different” and thus varies between the comparison groups, researchers cannot determine whether their data results from functional connectivity differences between these groups or from the differences in motions of their heads. Respiration undercuts the reliability of these datasets for addressing these same kinds of questions when researchers use one of the novel correction methods for head motion. Thus, both circumstances involve a “deviation” in data that does not result from the target factor or variable.

### Answering the framing question

With the case described, I return to the question framing this paper, inserting the case’s details: how did researchers end up with a second artifact, respiration, when trying to remediate the first artifact, head motion? The first blush response is that the novel remediation methods for head motion, introduced following the recognition of the inadequacy of realignment, changed data processing in a way that resulted in an artifact that is attributed to respiration.^8^ As I discussed in Sect. 2.2, remediation changes the experimental arrangement. Given that correction was used in this case, remediation affected more than just the relation between a head motion confounder and the dataset via a change to data processing factors.

There are two potential issues that might be at play in this scenario. First, the issue could be with the correction method. This intervention, intended to compensate only for head motion via changes to data processing factors, did not change these factors in a sufficiently *surgical* way as to only fulfill this intended aim. The identification of the second artifact, attributed to respiration, shows that other factors in the arrangement were changed. In essence, the remediation for head motion was “fat-handed” (Woodward, 2008), meaning that it affected several factors simultaneously, which resulted in an artifact.^9^

The second possible issue relates to the experimental arrangement itself. This arrangement, when modeled as a causal system, might not satisfy the criterion of *causal modularity*. This sense of ‘modularity,’ stemming from research on causal modeling, states that a system is modular if “there is a possible intervention on the dependent variable that changes only that equation while the other equations in the system remain unchanged” (Woodward, 2005, p. 329). Craver and Dan-Cohen note that artifacts can “arise from certain ‘non-modularities’ involving the intervention,” or “cases in which the intervention into the system for the purposes of doing the experiment fundamentally alters the functional relationships among the variables in the system” (2021, p. 12).

Applied to my case, causal modularity would be satisfied were there a possible intervention on only the relation between the targeted factor(s) and the dataset, leaving all other factors, representable as other equations in the system, unchanged. There might not be any possible (surgical) intervention on this experimental arrangement, due to the system not being causally modular. In essence, there might be no way to compensate for head motion via changes in data processing without affecting the processing of data related to respiration. It is, minimally, unclear whether there is such an intervention, and there is some defeasible reason to believe that there is not, given the similarities between head motion and respiration.

The issue of surgical intervention and fat-handedness leaves open that there might be an intervention that only changes the variable of interest, but minimally shows that the remediation in question is not it. The issue of causal modularity does not; if the system is not modular, there is not a possible surgical intervention. Nonetheless, both point to an answer to the question framing this paper. In either case, the remediation method is an intervention, but the consequence of this intervention is not a change to only the intended factor(s). Other factors of data production or processing can also be affected by this intervention, which can result in an artifact.

### What is needed to avoid the problem?

My appeal to surgical remediation methods and the causal modularity of the system do more than help to answer the question framing this paper. It also shows the limitations of diagnostic and determinative probes in the control of confounders. Before the start of the research depicted in my case, neuroimagers were aware that head motion and respiration are confounders in rs-fcMRI, and they were included in the confounder repertoire. A diagnostic probe would not have benefitted researchers using this experimental arrangement: they knew to look for these factors. Likewise, a determinative probe would not have helped either: researchers knew that these factors are present. These probes had been performed in the past, and the researchers thought they had controlled for them.

One could respond that determinative probes must be performed every time the “experimental situation” changes. Once the new head motion remediation methods were added to the experimental arrangement, the situation changed, warranting the probe to be performed again. I am sympathetic to this response, and it is consistent with Schickore’s claims that one’s confounder repertoire and probes are context specific. Any time the “experimental situation” (i.e., the experimental arrangement) changes, including any time a new remediation method is deployed, it is incumbent upon researchers to re-perform determinative probes.

However, this response does not undermine my claim about the limitation of a determinative probe in this case, as the very nature of correction entails that one has not eliminated the confounder from the experimental arrangement. This remediation method compensates for a factor that is still, in a real sense, muddling the relation between target and data (whence the need for compensation). Thus, it is unclear what information a determinative probe could provide in any situation in which correction is performed, and perhaps in situations where other remediation methods are used as well. Something else about the confounder must be learned in this situation, which goes beyond determining that it is present in the arrangement. Schickore might accept this conclusion, as she does not explicitly state that these probes are sufficient for ruling out confounders. I accept that these probes are valuable, if not necessary, for identifying confounders. Nonetheless, identifying a confounder need not resolve it. Thus, we should address what is needed for controlling confounders once they are identified.

The fact that we should not presuppose that a remediation method is surgical or the arrangement satisfies the criterion of causal modularity suggests that simply knowing *that* this method remediates an artifact might be insufficient for predicting its effect on the dataset. What is needed, I argue, is a *causal probe*, where the task is to figure out *how* remediation affects factors in the arrangement. Determining whether a remediation affects other factors in an arrangement is best achieved via determining how this remediation works. Merely knowing that the method remediates this artifact will limit causal reasoning about how factors that contribute to data production and processing, including the remediation, affect the dataset.

While the neuroimagers in this case knew about head motion and respiration confounders, the work of Power and colleagues shows how they muddle the experimental arrangement, and correspondingly how the remediation deployed to address them works, was not known (2012, 2020). The neuroimagers knew whether the correction methods achieved their aim, where the aim, narrowly construed, is to remediate head motion artifacts. However, it is in answering this “how” question that researchers studying functional connectivity via rs-fcMRI were limited, which helps to answer why their remediation resulted in a second artifact.

Causal reasoning supported researchers’ ability to explain how realignment affects data processing when it is applied in this experimental arrangement. They determined that realignment affects data processing, helping them to explain why this method remediates movement of the head through space but does not remediate disruptions of magnetic gradients. This example shows that researchers can causally probe into the confounders in an arrangement as well as the remediation. Learning that head motion is not merely present but also is multifaceted is needed for determining how this confounder ought to be remediated. Correspondingly, causally reasoning about remediation allows researchers to assess whether it will be effective. Because confounders and remediation are both factors in an arrangement, causal probes can be used to determine how these factors affect others. For this reason, causal probes aid in preventing a situation in which the remediation of one artifact results in another.

Focusing on the ability to causally reason about the relation between remediation and other factors of the experimental arrangement highlights that the answer to the question I have posed is not that remediation must be surgical or that the arrangement must satisfy the criterion of causal modularity. Rather, researchers should determine whether the intervention is surgical and the criterion is satisfied, and they should probe the causal relations between remediation and these factors if they are not. In other words, any one remediation method need not be surgical, and the system need not be causally modular.

My argument that any one remediation method need not be surgical and the arrangement need not satisfy the criterion of causal modularity might come across as unintuitive, so let me break down this argument in more detail. Surgical remediation methods and modular arrangements are patently desirable; if researchers do not alter any other part of the experimental arrangement when remediating, factors in the confounder repertoire will not result in a new artifact if they have already been controlled. This is most obvious for a surgical intervention: if there is one, researchers should try and use it. Likewise, researchers who erroneously think or presuppose that their remediation is surgical or the arrangement satisfies the criterion are liable to end up with an artifact, as this remediation might affect other factors, including confounders in the repertoire. This is my diagnosis of what happened in this case.

If researchers can determine that their remediation is not surgical or the arrangement does not satisfy the criterion, all is not lost. Instead of focusing on changing one factor via a single remediation method, they can adjust their set of remediation methods for the present artifact, for other factors in the arrangement, and for previously controlled artifacts stemming from factors in the confounder repertoire. Thus, no one intervention might be sufficient, but through an orchestration of changes, researchers can resolve the situation with a remediation of the artifact and no new one resulting in the process. This orchestration involves taking a holistic approach to remediation, where researchers do not aim to intervene on factors without considering the remainder of the arrangement. Rather than a piecemeal approach, researchers can account for the total set of changes that their remediation methods have. What is required for this orchestration is researchers knowing how their remediation methods affect the other factors of the arrangement and responding accordingly. This information is what is provided by a causal probe.

Given that I argue that information provided by a causal probe is what is required when remediating artifacts, my position stands in opposition to Feest’s account. I argue that researchers need not meet nor aim to meet the challenges that they have ensured that their manipulation only affects, or that their measure only measures, the relevant variable. I agree with Feest to an extent: ensuring these things is desirable, and researchers who erroneously think that they have devised their experimental arrangement to ensure these things are liable to end up with an artifact. Further, meeting the challenge that the manipulation only affects the relevant variable is consonant with prevention, while meeting the challenge that measures only measure the relevant variable is consonant with reduction. Both are viable methods for remediating artifacts.

However, these challenges need not be met. Ensuring these relations is not required to determine that researchers have adequate control in their arrangement, especially when researchers opt to retroactively correct for artifacts. This point is exemplified in my case. Researchers failing to meet these challenges is not what resulted in the problem that I address in this paper. Head motion can be detected during data production, so long as it is corrected for in data processing and the appropriate adjustments are made to the remediation of respiration and the other controlled factors in the confounder repertoire.

My defense of the requirement of causal probes supports one component of Schickore’s account, which I think is quite well-put, and I endorse. This component is the idea of the confounder repertoire. The idea that researchers are best off when they can causally reason about confounders and methods intended to remediate them, with this reasoning informed by causal probes, highlights that we should not think of the confounders present in an experimental arrangement in isolation. As ‘repertoire’ implies, we must causally reason about confounders in tandem and treat this repertoire holistically. Any remediation need not be solely tied to factors thought to be responsible for an artifact it is intended to remediate. It can causally relate to the arrangement and its factors, and my case is an example of this scenario.

## A future of artifact remediation

I have endeavored to answer why remediating one artifact might lead to another, and what this scenario tells us about artifacts, the factors responsible for them, and the ways in which researchers might try to control them. My answer highlights: (1) the importance of how remediation relates to the factors of an experimental arrangement, and (2) the benefits of a probe that supports causal reasoning about remediation when considering factors responsible for artifacts as a holistic repertoire rather than as singular, modular factors. In this section, I provide more defense for my position, and specifically for my appeal to the unique characteristics of correcting for artifacts, by drawing parallels between my position and a (probably quite near) future of artifact remediation across the experimental life sciences.

In my case, several of the updated head motion remediation methods, introduced as a response to the inadequacies of realignment, were automated correction methods. This provides me an avenue to relate my discussion of researchers’ (in)ability to causally reason about remediation to concerns about the transparency of complex computational systems. Specifically, my case parallels concerns about structural transparency, or “knowledge about how an algorithm is realized in code” (Creel, 2020, p. 575). This form of transparency captures whether researchers “understand how the code as written brings about the result of the program,” which reflects researchers’ knowledge of how the algorithm functions when it is used (Creel, 2020, p. 575).

The fact that the correction methods in my case had unpredicted consequences suggests that these methods, when included as factors of data processing, are structurally opaque. And, in being limited in their ability to reason about how their methods work, researchers did not predict that these correction methods would affect other factors of the experimental arrangement. Researchers knew that respiration could influence fMRI datasets, but they did not know that their correction methods would interfere with how respiration had been controlled for in the arrangement. Thus, due to what appears to be a result of structural opacity, researchers used these correction algorithms to fulfill their aims of removing head motion artifacts, but they inadvertently changed data processing, making it evidentially useless for studying their targets.

In framing this case as one involving structural opacity, I clarify what scientists do and do not know about their experimental arrangement when they use correction methods. Neuroimagers were in a better position to know whether the correction methods achieved their aim, where the aim, narrowly construed, is to remediate head motion artifacts. Thus, there is no comparable threat to functional transparency, or “knowledge of the algorithmic functioning of the whole” in this case (Creel, 2020, p. 573). However, knowing that an algorithm works is not equivalent to knowing how it works, consistent with my claims in Sect. 4.2.^10^

The parallels between my concerns about the need for adequate causal probes and literature on transparency provides a distinct lens with which we can discuss why remediating one artifact might lead to another when algorithmic correction is deployed. This lens is useful, as it affords me the opportunity for a speculation about the future of remediation across the experimental life sciences, which will help me to vindicate my position as well as my focus on correction.

Before I supply my speculation, let me provide some context for the scope of my claims in this paper. While my position is one that applies generally to artifact remediation in the life sciences, I admit that the specific desire to remediate via correction in my case stems at least in part from ethical considerations regarding the treatment of human subjects. Correction methods were deployed in this case in part because it is perceived to be unethical (fairly, in my opinion) to physically restrain the heads of children and individuals with neurodevelopmental disorders for the long times needed to scan for functional connectivity. It is likewise perceived to be unethical (again, I think fairly) to restrain their breathing with abdomen belts. Not all experiments in the life sciences have comparable ethical considerations, and for this reason prevention and reduction methods might be more commonly deployed in other areas.

However, ethical considerations are not the only reason correction methods might be deployed. As I mentioned in Sect. 2.2., correction, and specifically correction that is automated and algorithmic, might be much more convenient than other available options. These methods do not require researchers to fiddle with data production, making their manipulations or detectors sensitive to only the target. Instead, researchers can simply run studies as they have before and remediate artifacts on the back end of data processing, perhaps with an off-the-shelf correction tool that they can add to their data processing code. Indeed, if these correction methods work, why shouldn’t researchers use them instead of fiddling with other parts of an experimental arrangement? And, if the correction methods, fueled by the explosive advance of deep learning and computer vision, keep getting better and more convenient, what possible reason would dampen their increased use throughout the experimental life sciences?

With all this stated, I speculate that if attempts at applying new AI tools for correcting for artifacts has not already taken purchase across the experimental life sciences, then the only reason that these attempts will not be made in the very near future is if these tools radically alter experimental approaches in some way I cannot predict (perhaps they will so radically alter experimental paradigms as to render remediation perfunctory). This speculation is, I hope, a reasonable imaginary. It in turn provides reason for us to take seriously a future in which correction is the dominant form of remediating artifacts across the life sciences.

If this imaginary turns out to be the case, I am not naïve as to suggest that these tools will fail at correcting for artifacts; they are computationally powerful and advancing at a swift pace. My point, instead, is that using these tools to remediate one artifact still might result in another. This will not be due to a failure to meet the challenges of manipulation or measure described by Feest; rather, this can be for the same reason I have addressed throughout this paper. I predict that this outcome will occur if these tools are solely trained on data involving one artifact stemming from a particular confounder or only reinforced on correcting for this artifact. A myopic approach to the training—for instance, training an artificial neural network to search for and automatically correct signatures of head motion in fMRI data—likely will result in a correction method for head motion. However, even if it is powerful and sophisticated, this intervention still need not be surgical and the arrangement still need not satisfy the criterion of causal modularity. For this reason, the manipulation to data processing via correction might affect other factors in the arrangement, outside of its training parameters.

In this imaginary, it would aid researchers to know how any tool remediates when it does, so that they can determine the factors of the experimental arrangement that they are liable to affect. In other words, a causal probe is beneficial here, a case involving structural opacity, as it is in any case of remediation for the same reason that I addressed in Sect. 4.2. Likewise, treating a confounder repertoire holistically when training these tools and curating their training datasets, especially if the arrangement contains other remediation methods that have been inherited from previous iterations of the study, will help to avoid the issue for the same reason it would have helped in my case. With speculations about the future of artifact remediation in mind, we ought to develop accounts of artifacts and their control that are consistent with the viability of correction, which changes the arrangement by changing data processing.

## Conclusion

Philosophical interest in artifacts and the factors responsible for them has grown in the past few years, but these accounts must be paired with an analysis of remediation. In this paper, I have addressed how researchers remediate artifacts in their datasets, along with the challenges that they face in the process. The case of head motion in rs-fcMRI shows that, without using causal probes to learn about how confounders and remediation affect an experimental arrangement, researchers run the risk of remediating one artifact in a manner that results in another. My analysis highlights the limitations in how philosophers have construed artifacts and the factors responsible for them, particularly when we consider correction. These limitations become salient when we explore a sort of “failure” to remediate an artifact. Given that we do not want to reproduce these “failures” in the future, especially as a technological sea change is likely to bring with it an increased use of correction methods powered by tools like deep learning and computer vision, it is wise that we recognize how the nature of artifacts, factors that are responsible to them, and methods used to remediate them relate.

I conclude by reflecting on the difficulties that surround the remediation of artifacts. The reader might find my claims about causal reasoning to be too strong, as potentially countless factors can influence data production and processing in an experimental arrangement. Acquiring this knowledge, especially in arrangements where the criterion of causal modularity is not satisfied, seems like a Sisyphean task. In response to this concern, I agree that remediation does not depend on complete knowledge of all factors that might influence data. If a previously unidentified factor turns out to be a confounder out of the blue, then it may only be addressable following its discovery. However, many an artifact does not appear out of the blue, as if it were an “unconceived alternative” (Stanford, 2006). If researchers have reason to think a factor might result in an artifact, such as when a factor is in a confounder repertoire, they ought to know how this factor affects data and how remediation of an artifact relates to this factor.

## Footnotes

1 If one takes ‘intervention’ to be defined as a surgical manipulation, then one can substitute ‘manipulation’ whenever I talk of interventions that are not surgical.

2 Like Craver and Dan-Cohen (2021), I do not differentiate ‘artifact’ and ‘artefact.’ Like them, I attribute the use of two terms to American and British language conventions.

3 For a case where artifacts debatably are human creations, see Rasmussen, 1993 and Culp, 1994.

4 See Colaço, 2018 for a discussion of confounders (or confounds) as undercutting defeaters.

5 Craver and Dan-Cohen might reject head motion as an artifact source. For them, artifacts stem from “the experimental arrangement itself,” such as “the apparatus or procedure” (Craver & Dan-Cohen, 2021, p. 25). As they do not define ‘experimental arrangement,’ any disagreement might result from my inclusion of participant actions in an arrangement. What matters is that the remediation of error can bring about unintended consequences, whichever term is used.

6 Van Dijk and colleagues determined this by studying participant head motion differences (2012). Essentially, they studied head motion instead of age and neurodevelopmental disorders.

7 Like Schickore and Feest, I use ‘confounder’ informally, as it is sufficient to realize the problem that I address. However, I note that head motion is a candidate for a more technical account (VanderWeele & Shpitser, 2013), given that it co-varies with the variable of interest.

8 This is not a criticism of researchers’ actions. The case is ongoing, and the tribulations that they face are the steps they must take to learn how remediation affects a dataset. Even when restricting our discussion to identified factors, remediation is difficult. The tests researchers perform, such as those by Power and colleagues, typify what needed to remediate artifacts.

9 Indeed, there is a worry that psychological interventions are often fat-handed (Eronen, 2020).

10 This parallel is further exemplified by philosophers’ interests in artifacts resulting from complex computation systems more generally (Creel, 2020; Buckner, 2020).
